# *N*-Cyclo­hexyl-*N*-ethyl-4-methyl­benzene­sulfonamide

**DOI:** 10.1107/S1600536809052593

**Published:** 2009-12-12

**Authors:** Zeeshan Haider, Muhammad Nadeem Arshad, Jim Simpson, Islam Ullah Khan, Muhammad Shafiq

**Affiliations:** a Materials Chemistry Laboratory, Department of Chemistry, GC University, Lahore, Pakistan; b Department of Chemistry, University of Otago, PO Box 56, Dunedin, New Zealand

## Abstract

The title compound, C_15_H_23_NO_2_S, contains cyclo­hexyl and ethyl substituents on the sulfonamide N atom and the cyclo­hexyl ring adopts a classic chair conformation. The dihedral angle between the benzene ring plane and the mean plane through the six atoms of the cyclo­hexyl ring is 59.92 (6)°. In the crystal structure, C—H⋯O hydrogen bonds link mol­ecules into sheets extending in the *bc* plane.

## Related literature

For ring conformations, see: Cremer & Pople (1975 ▶). For related structures, see: Arshad *et al.* (2008 ▶, 2009 ▶); Khan *et al.* (2009 ▶); Gowda *et al.* (2007*a* ▶,*b* ▶,*c* ▶).
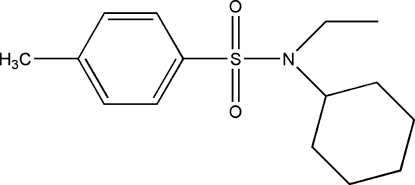


## Experimental

### 

#### Crystal data

C_15_H_23_NO_2_S
*M* *_r_* = 281.40
Monoclinic, 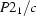

*a* = 12.2269 (5) Å
*b* = 7.5818 (3) Å
*c* = 16.3045 (6) Å
β = 92.495 (2)°
*V* = 1510.03 (10) Å^3^
*Z* = 4
Mo *K*α radiation
μ = 0.21 mm^−1^
*T* = 296 K
0.43 × 0.32 × 0.15 mm

#### Data collection

Bruker APEXII CCD area-detector diffractometer
Absorption correction: multi-scan (*SADABS*; Bruker, 2007 ▶) *T*_min_ = 0.914, *T*_max_ = 0.969
16676 measured reflections
3714 independent reflections
2251 reflections with *I* > 2σ(*I*)
*R*_int_ = 0.040

#### Refinement

*R*[*F* ^2^ > 2σ(*F* ^2^)] = 0.045
*wR*(*F* ^2^) = 0.122
*S* = 0.99
3713 reflections
174 parameters
H-atom parameters constrained
Δρ_max_ = 0.23 e Å^−3^
Δρ_min_ = −0.25 e Å^−3^

### 

Data collection: *APEX2* (Bruker, 2007 ▶); cell refinement: *SAINT* (Bruker, 2007 ▶); data reduction: *SAINT*; program(s) used to solve structure: *SHELXS97* (Sheldrick, 2008 ▶); program(s) used to refine structure: *SHELXL97* (Sheldrick, 2008 ▶); molecular graphics: *ORTEP-3* (Farrugia, 1997 ▶) and *Mercury* (Macrae *et al.*, 2006 ▶); software used to prepare material for publication: *SHELXL97*, *enCIFer* (Allen *et al.*, 2004 ▶)),  *PLATON* (Spek, 2009 ▶) and *publCIF* (Westrip, 2009 ▶).

## Supplementary Material

Crystal structure: contains datablocks global, I. DOI: 10.1107/S1600536809052593/bt5132sup1.cif

Structure factors: contains datablocks I. DOI: 10.1107/S1600536809052593/bt5132Isup2.hkl

Additional supplementary materials:  crystallographic information; 3D view; checkCIF report

## Figures and Tables

**Table 1:** Hydrogen-bond geometry (Å, °)

*D*—H⋯*A*	*D*—H	H⋯*A*	*D*⋯*A*	*D*—H⋯*A*
C10—H10*B*⋯O2^i^	0.97	2.66	3.530 (3)	150
C13—H13*A*⋯O1^ii^	0.96	2.60	3.512 (3)	159

Symmetry codes: (i) 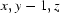; (ii) 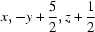.

**Fig. 1.**
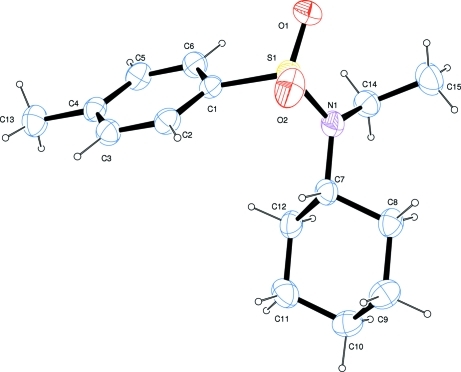
The structure of (I) with displacement ellipsoids for the non-hydrogen atoms drawn at the 50% probability level.

**Fig. 2.**
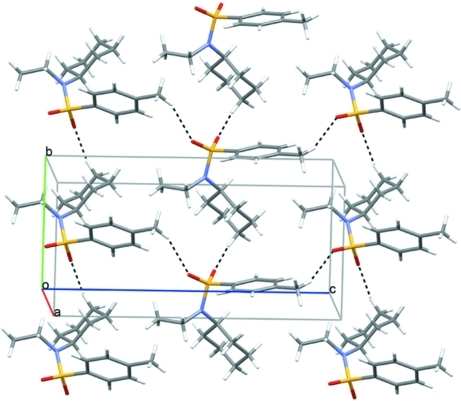
Crystal packing of (I) viewed down the a axis with hydrogen bonds drawn as dashed lines.

## supplementary crystallographic information

### Comment

Our group has been involved in the synthesis and crystallographic studies of sulfonamide derivatives (Arshad *et al.*, 2009).

The title compound is a benzenesulfonamide with cyclohexyl and ethyl substituents on the sulfonamide N1 atom, Fig. 1. Bond distances in the molecule are   comparable to those in similar structures (Arshad *et al.*, 2008; Khan *et al.*, 2009; Gowda *et al.*, 2007*a*,*b*,*c*). The cyclohexyl (C7···C12) ring adopts a classic chair conformation with puckering amplitude Q = 0.567 (2) Å, θ = 178.8 (2) °, φ = 214 (11) ° (Cremer & Pople, 1975) and the plane through this ring is inclined at 59.92 (6) ° to that of the C1···C6 benzene ring. In the crystal structure C—H···O hydrogen bonds involving the C13–H13 bond of the methyl group and the C10–H10B bond of the cyclohexyl ring link each molecule to the O1 and O2 atoms of individual sulphonamide units forming an extended two dimensional network in the *bc* plane (Table 1, Fig. 2).

### Experimental

A mixture of *N*-cyclohexyl-4-methyl benzene sulfonamide (1.089 g, 4.3 mmol), sodium hydride (0.21 g, 8.6 mmol) and N, *N*-dimethylformamide (10 ml) was stirred at room temperature for half an hour followed by addition of ethyl iodode (1.32 g, 8.6 mmol). Stirring was continued further for a period of three hours and the contents were poured over crushed ice. Precipitated product was isolated, washed and crystallized from a methanol solution.

### Refinement

All H-atoms were positioned geometrically and refined using a riding model with d(C—H) = 0.93 Å, *U*_iso_=1.2*U*_eq_ (C) for aromatic 0.98 Å, *U*_iso_ = 1.2*U*_eq_ (C) for CH, 0.97 Å, *U*_iso_ = 1.2*U*_eq_ (C) for CH_2_, 0.96 Å, *U*_iso_ = 1.5*U*_eq_ (C) for CH_3_ atoms.

The 1 0 0 reflection was identified as being obscured by the beamstop and was omitted.

### Crystal data

**Table:**

C_15_H_23_NO_2_S	*F*(000) = 608
*M**_r_* = 281.40	*D*_x_ = 1.238 Mg m^−^^3^
Monoclinic, *P*2_1_/*c*	Mo *K*α radiation, λ = 0.71073 Å
Hall symbol: -P 2ybc	Cell parameters from 3495 reflections
*a* = 12.2269 (5) Å	θ = 2.5–25.6°
*b* = 7.5818 (3) Å	µ = 0.21 mm^−^^1^
*c* = 16.3045 (6) Å	*T* = 296 K
β = 92.495 (2)°	Needle, white
*V* = 1510.03 (10) Å^3^	0.43 × 0.32 × 0.15 mm
*Z* = 4	

### Data collection

**Table:**

Bruker APEXII CCD area-detector diffractometer	3714 independent reflections
Radiation source: fine-focus sealed tube	2251 reflections with *I* > 2σ(*I*)
graphite	*R*_int_ = 0.040
φ and ω scans	θ_max_ = 28.3°, θ_min_ = 1.7°
Absorption correction: multi-scan (*SADABS*; Bruker, 2007)	*h* = −16→15
*T*_min_ = 0.914, *T*_max_ = 0.969	*k* = −10→9
16676 measured reflections	*l* = −19→21

### Refinement

**Table:**

Refinement on *F*^2^	Primary atom site location: structure-invariant direct methods
Least-squares matrix: full	Secondary atom site location: difference Fourier map
*R*[*F*^2^ > 2σ(*F*^2^)] = 0.045	Hydrogen site location: inferred from neighbouring sites
*wR*(*F*^2^) = 0.122	H-atom parameters constrained
*S* = 0.99	*w* = 1/[σ^2^(*F*_o_^2^) + (0.048*P*)^2^ + 0.3506*P*] where *P* = (*F*_o_^2^ + 2*F*_c_^2^)/3
3713 reflections	(Δ/σ)_max_ = 0.001
174 parameters	Δρ_max_ = 0.23 e Å^−^^3^
0 restraints	Δρ_min_ = −0.25 e Å^−^^3^

### Special details

**Table:**

Geometry. All s.u.'s (except the s.u. in the dihedral angle between two l.s. planes) are estimated using the full covariance matrix. The cell s.u.'s are taken into account individually in the estimation of s.u.'s in distances, angles and torsion angles; correlations between s.u.'s in cell parameters are only used when they are defined by crystal symmetry. An approximate (isotropic) treatment of cell s.u.'s is used for estimating s.u.'s involving l.s. planes.
Refinement. Refinement of *F*^2^ against ALL reflections. The weighted *R*-factor *wR* and goodness of fit *S* are based on *F*^2^, conventional *R*-factors *R* are based on *F*, with *F* set to zero for negative *F*^2^. The threshold expression of *F*^2^ > 2σ(*F*^2^) is used only for calculating *R*-factors(gt) *etc*. and is not relevant to the choice of reflections for refinement. *R*-factors based on *F*^2^ are statistically about twice as large as those based on *F*, and *R*- factors based on ALL data will be even larger.

### Fractional atomic coordinates and isotropic or equivalent isotropic displacement parameters (Å^2^)

**Table:**

	*x*	*y*	*z*	*U*_iso_*/*U*_eq_	
S1	0.22960 (5)	1.10442 (7)	0.56680 (3)	0.05930 (19)	
O1	0.14930 (14)	1.1507 (2)	0.50392 (9)	0.0866 (5)	
O2	0.32184 (13)	1.21640 (19)	0.58303 (10)	0.0784 (5)	
N1	0.27372 (13)	0.9101 (2)	0.54440 (9)	0.0549 (4)	
C1	0.16215 (14)	1.0870 (2)	0.66002 (11)	0.0451 (4)	
C2	0.21516 (14)	1.1373 (2)	0.73271 (11)	0.0511 (5)	
H2	0.2856	1.1835	0.7326	0.061*	
C3	0.16309 (15)	1.1186 (3)	0.80559 (12)	0.0528 (5)	
H3	0.1994	1.1519	0.8544	0.063*	
C4	0.05847 (15)	1.0517 (2)	0.80760 (11)	0.0491 (5)	
C5	0.00638 (16)	1.0042 (3)	0.73394 (13)	0.0571 (5)	
H5	−0.0645	0.9598	0.7341	0.069*	
C6	0.05660 (16)	1.0209 (3)	0.66051 (12)	0.0555 (5)	
H6	0.0201	0.9881	0.6117	0.067*	
C7	0.36213 (15)	0.8297 (2)	0.59696 (11)	0.0494 (5)	
H7	0.3938	0.9237	0.6317	0.059*	
C8	0.45338 (16)	0.7582 (3)	0.54568 (12)	0.0576 (5)	
H8A	0.4245	0.6660	0.5096	0.069*	
H8B	0.4808	0.8521	0.5118	0.069*	
C9	0.54647 (16)	0.6847 (3)	0.59987 (14)	0.0671 (6)	
H9A	0.6019	0.6358	0.5657	0.081*	
H9B	0.5797	0.7793	0.6323	0.081*	
C10	0.50692 (17)	0.5438 (3)	0.65621 (13)	0.0642 (6)	
H10A	0.5673	0.5044	0.6922	0.077*	
H10B	0.4811	0.4435	0.6239	0.077*	
C11	0.4154 (2)	0.6108 (3)	0.70741 (13)	0.0697 (6)	
H11A	0.4437	0.7014	0.7446	0.084*	
H11B	0.3883	0.5148	0.7402	0.084*	
C12	0.32174 (16)	0.6864 (3)	0.65394 (12)	0.0606 (5)	
H12A	0.2670	0.7355	0.6887	0.073*	
H12B	0.2876	0.5926	0.6214	0.073*	
C13	0.00216 (18)	1.0322 (3)	0.88731 (13)	0.0723 (6)	
H13A	0.0434	1.0933	0.9300	0.108*	
H13B	−0.0027	0.9095	0.9011	0.108*	
H13C	−0.0701	1.0815	0.8817	0.108*	
C14	0.21331 (17)	0.8024 (3)	0.48246 (12)	0.0608 (5)	
H14A	0.1357	0.8263	0.4855	0.073*	
H14B	0.2249	0.6787	0.4953	0.073*	
C15	0.2469 (2)	0.8363 (3)	0.39659 (13)	0.0820 (7)	
H15A	0.2338	0.9578	0.3828	0.123*	
H15B	0.2050	0.7624	0.3592	0.123*	
H15C	0.3233	0.8104	0.3927	0.123*	

### Atomic displacement parameters (Å^2^)

**Table:**

	*U*^11^	*U*^22^	*U*^33^	*U*^12^	*U*^13^	*U*^23^
S1	0.0778 (4)	0.0485 (3)	0.0529 (3)	0.0114 (3)	0.0175 (2)	0.0068 (2)
O1	0.1182 (13)	0.0855 (12)	0.0565 (9)	0.0423 (10)	0.0071 (9)	0.0196 (8)
O2	0.0959 (11)	0.0491 (9)	0.0935 (11)	−0.0132 (8)	0.0419 (9)	−0.0031 (8)
N1	0.0682 (10)	0.0513 (10)	0.0455 (9)	0.0081 (8)	0.0054 (7)	−0.0054 (8)
C1	0.0487 (10)	0.0398 (10)	0.0470 (10)	0.0075 (8)	0.0052 (8)	0.0001 (8)
C2	0.0410 (10)	0.0532 (12)	0.0591 (12)	−0.0008 (8)	0.0027 (9)	−0.0036 (9)
C3	0.0498 (11)	0.0590 (12)	0.0493 (11)	0.0003 (9)	−0.0015 (8)	−0.0077 (9)
C4	0.0537 (11)	0.0404 (10)	0.0537 (11)	0.0041 (8)	0.0078 (9)	−0.0022 (9)
C5	0.0451 (11)	0.0574 (13)	0.0692 (14)	−0.0064 (9)	0.0069 (9)	−0.0074 (10)
C6	0.0544 (12)	0.0569 (12)	0.0545 (12)	−0.0010 (10)	−0.0049 (9)	−0.0104 (10)
C7	0.0611 (11)	0.0441 (10)	0.0433 (10)	0.0015 (9)	0.0046 (8)	−0.0062 (8)
C8	0.0590 (12)	0.0559 (12)	0.0593 (12)	−0.0029 (10)	0.0164 (9)	0.0029 (10)
C9	0.0551 (12)	0.0620 (14)	0.0845 (16)	−0.0010 (11)	0.0058 (11)	−0.0003 (12)
C10	0.0640 (13)	0.0517 (12)	0.0760 (15)	0.0047 (10)	−0.0062 (11)	−0.0023 (11)
C11	0.0958 (16)	0.0593 (14)	0.0543 (12)	0.0046 (12)	0.0053 (11)	0.0072 (11)
C12	0.0674 (13)	0.0616 (13)	0.0543 (12)	0.0078 (11)	0.0207 (10)	0.0053 (10)
C13	0.0765 (15)	0.0772 (16)	0.0649 (14)	−0.0025 (12)	0.0229 (11)	−0.0008 (12)
C14	0.0638 (12)	0.0607 (13)	0.0576 (12)	−0.0026 (10)	−0.0001 (10)	−0.0015 (10)
C15	0.0992 (18)	0.0938 (19)	0.0525 (13)	−0.0011 (15)	−0.0013 (12)	−0.0099 (13)

### Geometric parameters (Å, °)

**Table:**

S1—O2	1.4272 (16)	C8—H8B	0.9700
S1—O1	1.4320 (16)	C9—C10	1.502 (3)
S1—N1	1.6160 (17)	C9—H9A	0.9700
S1—C1	1.7654 (18)	C9—H9B	0.9700
N1—C14	1.472 (2)	C10—C11	1.513 (3)
N1—C7	1.481 (2)	C10—H10A	0.9700
C1—C2	1.380 (2)	C10—H10B	0.9700
C1—C6	1.385 (3)	C11—C12	1.521 (3)
C2—C3	1.380 (3)	C11—H11A	0.9700
C2—H2	0.9300	C11—H11B	0.9700
C3—C4	1.378 (3)	C12—H12A	0.9700
C3—H3	0.9300	C12—H12B	0.9700
C4—C5	1.383 (3)	C13—H13A	0.9600
C4—C13	1.504 (3)	C13—H13B	0.9600
C5—C6	1.375 (3)	C13—H13C	0.9600
C5—H5	0.9300	C14—C15	1.498 (3)
C6—H6	0.9300	C14—H14A	0.9700
C7—C8	1.523 (3)	C14—H14B	0.9700
C7—C12	1.525 (3)	C15—H15A	0.9600
C7—H7	0.9800	C15—H15B	0.9600
C8—C9	1.517 (3)	C15—H15C	0.9600
C8—H8A	0.9700		
			
O2—S1—O1	119.90 (11)	C8—C9—H9A	109.4
O2—S1—N1	108.38 (9)	C10—C9—H9B	109.4
O1—S1—N1	106.68 (9)	C8—C9—H9B	109.4
O2—S1—C1	106.25 (9)	H9A—C9—H9B	108.0
O1—S1—C1	107.65 (9)	C9—C10—C11	111.34 (18)
N1—S1—C1	107.42 (8)	C9—C10—H10A	109.4
C14—N1—C7	120.00 (16)	C11—C10—H10A	109.4
C14—N1—S1	119.89 (13)	C9—C10—H10B	109.4
C7—N1—S1	119.14 (12)	C11—C10—H10B	109.4
C2—C1—C6	119.88 (17)	H10A—C10—H10B	108.0
C2—C1—S1	119.95 (14)	C10—C11—C12	111.52 (17)
C6—C1—S1	120.16 (15)	C10—C11—H11A	109.3
C3—C2—C1	119.63 (17)	C12—C11—H11A	109.3
C3—C2—H2	120.2	C10—C11—H11B	109.3
C1—C2—H2	120.2	C12—C11—H11B	109.3
C2—C3—C4	121.47 (17)	H11A—C11—H11B	108.0
C2—C3—H3	119.3	C11—C12—C7	111.20 (17)
C4—C3—H3	119.3	C11—C12—H12A	109.4
C3—C4—C5	117.93 (17)	C7—C12—H12A	109.4
C3—C4—C13	121.15 (18)	C11—C12—H12B	109.4
C5—C4—C13	120.93 (18)	C7—C12—H12B	109.4
C6—C5—C4	121.74 (18)	H12A—C12—H12B	108.0
C6—C5—H5	119.1	C4—C13—H13A	109.5
C4—C5—H5	119.1	C4—C13—H13B	109.5
C5—C6—C1	119.34 (18)	H13A—C13—H13B	109.5
C5—C6—H6	120.3	C4—C13—H13C	109.5
C1—C6—H6	120.3	H13A—C13—H13C	109.5
N1—C7—C8	111.26 (15)	H13B—C13—H13C	109.5
N1—C7—C12	113.46 (16)	N1—C14—C15	113.33 (17)
C8—C7—C12	110.21 (16)	N1—C14—H14A	108.9
N1—C7—H7	107.2	C15—C14—H14A	108.9
C8—C7—H7	107.2	N1—C14—H14B	108.9
C12—C7—H7	107.2	C15—C14—H14B	108.9
C9—C8—C7	111.12 (16)	H14A—C14—H14B	107.7
C9—C8—H8A	109.4	C14—C15—H15A	109.5
C7—C8—H8A	109.4	C14—C15—H15B	109.5
C9—C8—H8B	109.4	H15A—C15—H15B	109.5
C7—C8—H8B	109.4	C14—C15—H15C	109.5
H8A—C8—H8B	108.0	H15A—C15—H15C	109.5
C10—C9—C8	111.37 (17)	H15B—C15—H15C	109.5
C10—C9—H9A	109.4		
			
O2—S1—N1—C14	−144.40 (15)	C13—C4—C5—C6	−179.92 (19)
O1—S1—N1—C14	−14.01 (17)	C4—C5—C6—C1	0.0 (3)
C1—S1—N1—C14	101.19 (15)	C2—C1—C6—C5	−0.8 (3)
O2—S1—N1—C7	46.86 (16)	S1—C1—C6—C5	178.40 (15)
O1—S1—N1—C7	177.24 (14)	C14—N1—C7—C8	60.5 (2)
C1—S1—N1—C7	−67.56 (15)	S1—N1—C7—C8	−130.77 (15)
O2—S1—C1—C2	−15.54 (18)	C14—N1—C7—C12	−64.5 (2)
O1—S1—C1—C2	−145.16 (16)	S1—N1—C7—C12	104.27 (17)
N1—S1—C1—C2	100.29 (16)	N1—C7—C8—C9	177.02 (16)
O2—S1—C1—C6	165.26 (15)	C12—C7—C8—C9	−56.2 (2)
O1—S1—C1—C6	35.64 (18)	C7—C8—C9—C10	56.7 (2)
N1—S1—C1—C6	−78.91 (16)	C8—C9—C10—C11	−55.7 (2)
C6—C1—C2—C3	1.0 (3)	C9—C10—C11—C12	55.0 (2)
S1—C1—C2—C3	−178.17 (14)	C10—C11—C12—C7	−55.1 (2)
C1—C2—C3—C4	−0.5 (3)	N1—C7—C12—C11	−179.00 (15)
C2—C3—C4—C5	−0.3 (3)	C8—C7—C12—C11	55.5 (2)
C2—C3—C4—C13	−179.85 (19)	C7—N1—C14—C15	−103.9 (2)
C3—C4—C5—C6	0.6 (3)	S1—N1—C14—C15	87.5 (2)

### Hydrogen-bond geometry (Å, °)

**Table:**

*D*—H···*A*	*D*—H	H···*A*	*D*···*A*	*D*—H···*A*
C10—H10B···O2^i^	0.97	2.66	3.530 (3)	150
C13—H13A···O1^ii^	0.96	2.60	3.512 (3)	159

Symmetry codes: (i) *x*, *y*−1, *z*; (ii) *x*, −*y*+5/2, *z*+1/2.
